# Novel homozygous variant in the *TPO* gene associated with congenital hypothyroidism and mild-intellectual disability

**DOI:** 10.1038/s41439-020-00129-3

**Published:** 2020-11-27

**Authors:** Amjad Khan, Muhammad Umair, Rania Abdulfattah Sharaf, Muhammad Ismail Khan, Amir Ullah, Safdar Abbas, Nargis Shaheen, Muhammad Bilal, Farooq Ahamd

**Affiliations:** 1 Medical Genomics Research Department, King Abdullah International Medical Research Center (KAIMRC), King Saud bin Abdulaziz University for Health Science, Ministry of National Guard-Health Affairs (MNGHA), Riyadh, Saudi Arabia; 2 Department of Speech Language Pathology and Audiology, National Guard Health Affairs, Ministry of National Guard, Riyadh, Saudi Arabia; 3 Department of Zoology, Islamia College University, Peshawar, Pakistan; 4 Nephrology and Dialysis Unit, District Head Quarter (DHQ) Teaching Hospital, Bannu, 2800 Pakistan; 5 Department of Biochemistry, Faculty of Biological Sciences, Quaid-i-Azam University, Islamabad, Pakistan; 6 Department of Zoology, Faculty of Biological Sciences, Quaid-i-Azam University, Islamabad, Pakistan; 7 Department of Chemistry, Swabi University, KPK, Pakistan

**Keywords:** Consanguinity, Genomics

## Abstract

Congenital hypothyroidism (CH) is one of the most common hereditary disorders affecting neonates worldwide. CH is a multifactorial complex disorder and can be caused by either environmental factors or genetic factors. We studied one Pakistani family with segregating mutations in CH inherited in an autosomal recessive manner. Using whole-exome sequencing (WES), we found a novel homozygous missense variant (c.2315A>G; p.Tyr772Cys) in the thyroid peroxidase (TPO) gene. Different bioinformatics prediction tools and Sanger sequencing were performed to verify the identified variant. Our findings highlight the importance of this gene in causing CH and mild-intellectual disability (ID) in two affected brothers. WES is a convenient and useful tool for the clinical diagnosis of CH and other associated disorders.

## Introduction

Congenital hypothyroidism (CH) is diagnosed as thyroid hormone deficiency in newborn infants with an incidence of 1:2000 to 1:4000 live births worldwide^1^. Approximately 80–85% of CH cases are associated with thyroid dysgenesis (TD); either the thyroid gland is absent, reduced in size or versatile^2^. Upon the diagnosis of CH, early TH administration is a key to avoid severe structural, motor, and neurodevelopmental defects^3^. Molecular investigations assist in definitive diagnosis and precise classification of CH and might illustrate patient-specific targets for alternative treatment of the disease. Currently, there are a handful of genes known to be responsible for CH associated with both primary thyroid dysgenesis and thyroid dyshormonogenesis (TDH)^4^.

**Fig. 1:**
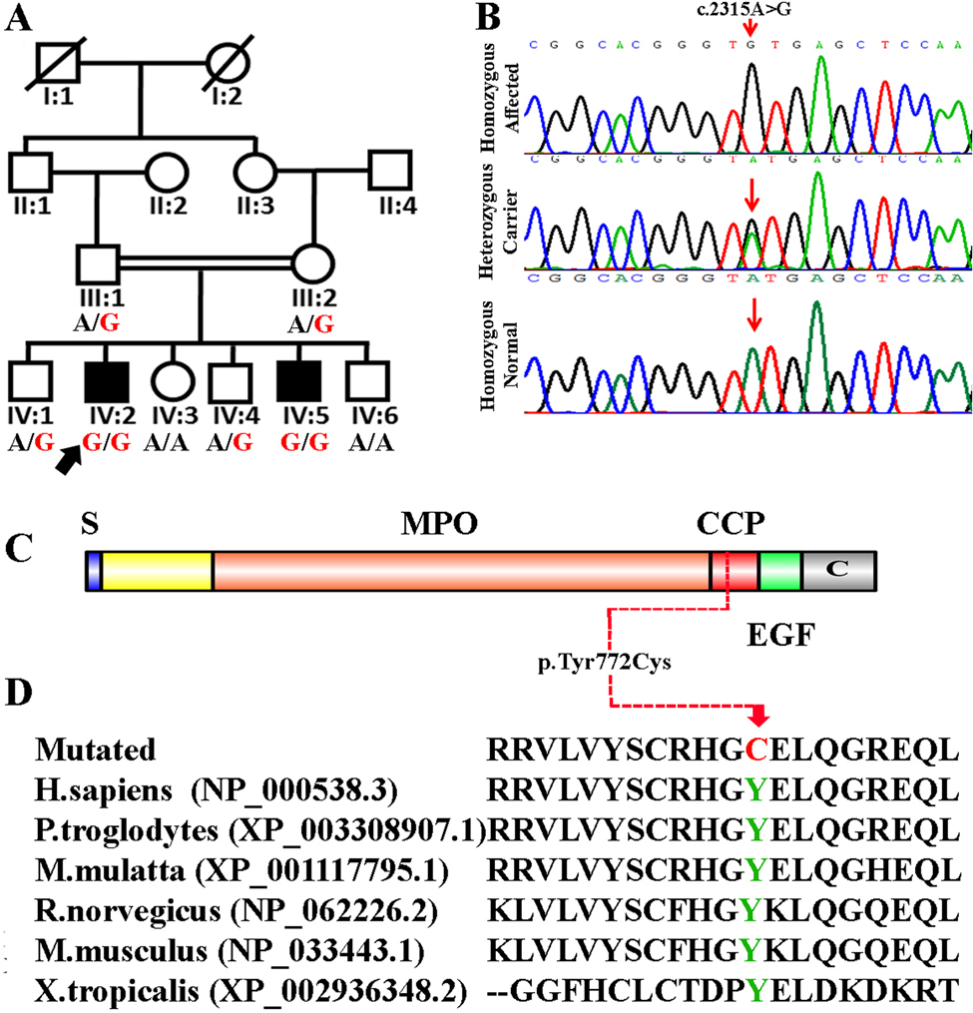
Genetic and molecular analysis. **A** A consanguineous pedigree showing two affected members (IV: 2 and IV: 5) in the fourth generation with congenital hypothyroidism. Affected individuals in the pedigree are shown with shaded symbols, and unaffected individuals are indicated with open symbols. Double lines indicate a consanguineous union. **B** Sequence chromatogram showing the missense *TPO* variant. The red arrow indicates the site of the identified variant in this study. Affected individuals (IV: 2 and IV: 5) are homozygous, parents (III: 1 and III: 2) and two normal brothers (IV: 1 and IV: 4) are heterozygous, and two siblings (IV: 3 and IV: 6) are homozygous normal for the identified variant. **C** Schematic representation of *TPO* protein and its domains; arrow indicates the location of the mutation identified in the present study. **D** Conservation of p.Tyr772 across several species.

In this report, we describe a consanguineous Pakistani family with two affected individuals (IV: 2, IV: 5) with CH and ID. Signed informed consent for the genetic analysis and publication of data was obtained from the patient’s legal guardians. A pedigree was generated (Fig. 1A), and the affected individuals were thoroughly examined by a local endocrinologist and geneticist. Index patient IV: 2 was diagnosed with CH and mild ID at the age of 26 when he underwent a thorough examination for prolonged jaundice. At the age of 26 years, patient IV: 2 had tall forehead, thick eyebrows, deep-set eyes, strabismus, thick lips, protruding ears, and a prominent goiter. He had already undergone thyroidectomy twice at the age of 11 and 20 years. The goiter size has gradually increased over the last few years. Family history revealed a brother (IV: 5) with CH and mild ID (IQ score 54) diagnosed at the age of 24. Patient IV: 5 had a prominent supraorbital ridge, with mild nasal flaring, no bulbous nasal tip, deep-set eyes, thick upper and lower lip, and pointed chin. He had normal ears and a short forehead. Hypoplastic philtrum, social and behavioral abnormalities, prominent goiter, ID (IQ: 54), and squint eye were noted. Further endocrine and laboratory workups showed thyroxin (T4) 1.50 µg/dl, free T4 0.4 ng/dl, triiodothyronine (T3) 7.4 ng/ul, thyroid stimulating hormone (TSH) 62 µU/ml, thyroglobulin (TG) 5.1 ng/ml, and thyroxine-binding globulin (TBG) 23 µg/ml.

For molecular investigation, fresh blood samples were drawn from the affected and normal siblings and parents. Genomic DNA was extracted using the QIAquick DNA Extraction Kit (Qiagen, Hilden, Germany). Whole-exome sequencing (WES) and data analysis were performed as described previously^5,6^. All variants were screened according to the location, frequency, and type of mutation (Supplementary Tables 1 & 2). We also focused on 21 known genes implicated in CH (Supplementary Table 3) and only found a novel homozygous missense variant (c.2315A>G; p.Tyr772Cys) in the *TPO* (NM_175719.3; rs1382787497) gene in both affected individuals (IV: 2 and IV: 5), which was confirmed by Sanger sequencing (Fig. 1B). The parents (III: 1 and III: 2) and two normal brothers (IV: 1 and IV: 4) were heterozygous, and the two siblings (IV: 3 and IV: 6) were homozygous normal for this variant (Fig. 1B).

Different bioinformatics tools, including Mutation Taster (http://www.mutationtaster.org/), Polyphen-2 (http://genetics.bwh.harvard.edu/pph2), Sorting Intolerant From Tolerant (SIFT, http://www.sift.jcvi.org/), Exome Sequencing Project (ESP, http://evs.gs.washington.edu/EVS/), Protein Variation Effect Analyzer (PROVEAN, http://www.provean.jcvi.org), Human Splicing Finder (HSF, http://www.umd.be/HSF/), Combined Annotation Dependent Depletion (CADD, https://cadd.gs.washington.edu/) and Varsome (https://varsome.com/), were used for functional effect prediction. The variant (c.2315A>G; p.Tyr772Cys) was also analyzed in 200 ethnically matched control individuals and 145 in-house (Pakistani) exomes. Finally, the American College of Medical Genetics and Genomics (ACMG) 2015 criteria and guidelines (PM2, PP3, PP2, PP1, and PP4) were used for the interpretation of variants that were classified as likely pathogenic^7^. The identified variant is located in a highly conserved complement control protein (CCP)-like domain in the *TPO* gene, which might affect the secondary structure and binding to other important proteins involved in the proper function of *TPO* (Fig. 1C, D). The *TPO* gene is located on chromosome 2p25 and has 17 exons, consisting of 150 kb of DNA and encodes a 933 amino acid-TPO enzyme.

**Fig. 2:**
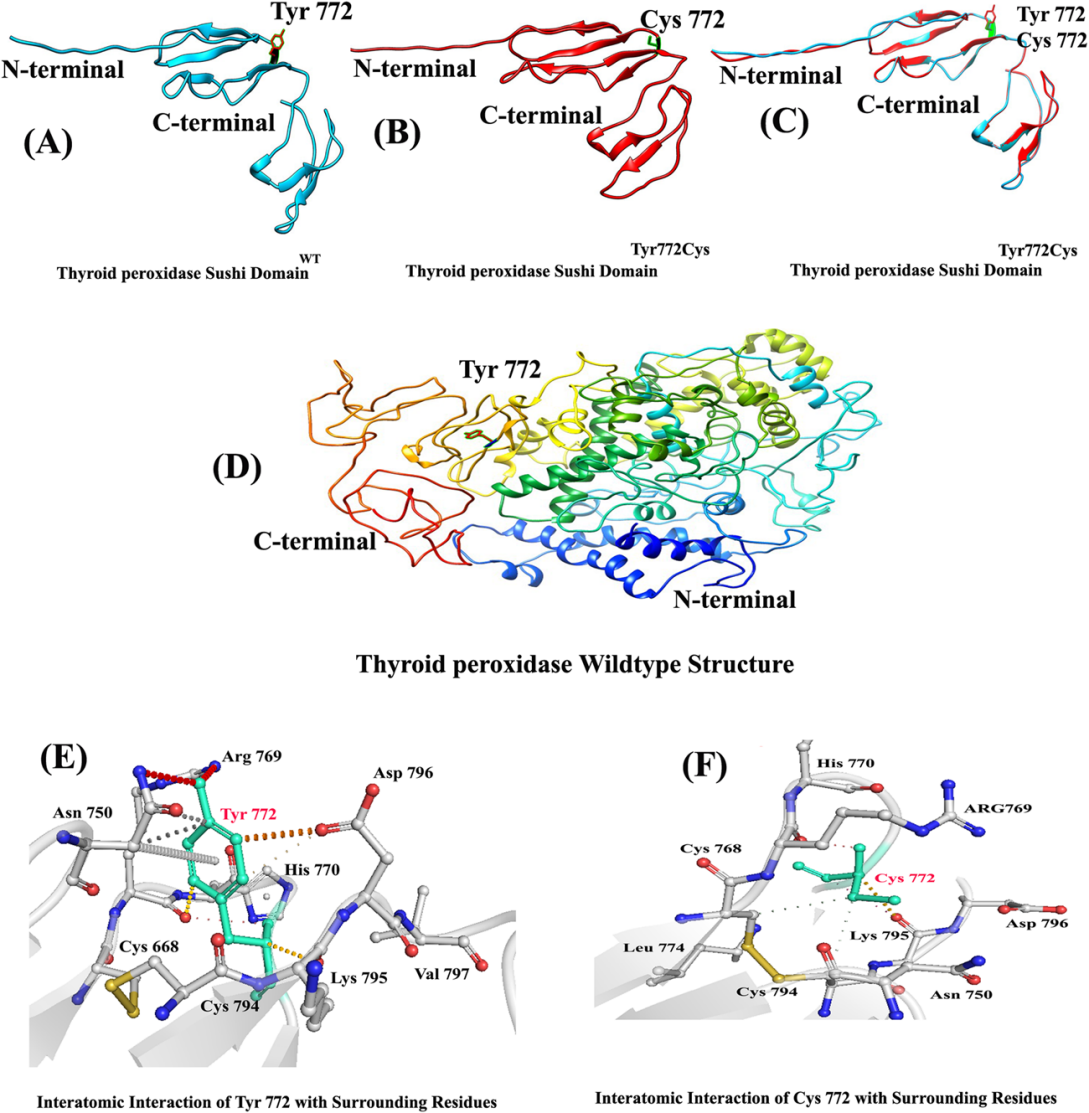
TPO protein 3D modeling. **A** *TPO* wild-type sushi domain, **B** *TPO* mutant-type sushi domain, **C** simulation of wild-type and mutant sushi domains of *TPO*, **D** TPO wild-type structure, **E** interaction of wild-type Tyr772 with its surrounding residues, and **F** interaction of mutant Cys 772 with its surrounding residues.

The crystal structure resolved at 1.99 Å resolution (PDB ID: 3ZD2)^8^ was used for the wild-type and mutant model structure and analysis. The amino acid (993 aa) sequence of the *TPO*-encoding protein was retrieved from the UniProt database with accession number P07202-1 in FASTA format. Structure visualization, measurement of distance, and mutagenesis analysis were performed with different bioinformatics software programs as described previously^5,9^. Our analysis revealed that Tyr772 interacts with Asn750, Cys668, His770, Arg769, Lys795, and Asp 796 (Fig. 2A–F). Tyrosine is an aromatic polar amino acid, but substitution of a smaller cysteine to a larger tyrosine disrupts its interaction with surrounding amino acid residues, and these new interactions in turn might potentially disrupt both protein secondary structure and function. Using DUET, ENCoM, SDM, and mCSM, we predicted that the Tyr772Cys mutation would cause a −0.585, −0.502, −1.28, and −0.54 kcal/mole change in the ΔΔ*G*, respectively, indicating that the mutation would greatly destabilize the protein structure and hence disrupt function.

To date, ~161 mutations (missense, nonsense, splice site and frameshift) associated with CH phenotypes have been described in the human gene mutation database (HGMD; http://www.hgmd.cf.ac.uk/ac/index.php) (Supplementary Table 4). Cangül et al.^10^ studied a consanguineous Turkish family with a homozygous nonsense mutation (c.1618C>T; p. p.R540X) in the *TPO* gene, leading to CH^11^. Another study on two Amish families by Pannain et al. identified homozygous missense mutations (c.2395G>A; p.Glu799Lys, and c.1943G>A; p.Arg648Gln) in the *TPO* gene with CH^12^. Fu et al.^1^ examined the *TPO* mutation spectrum and prevalence among 192 patients with CH in the Guangxi Zhuang Autonomous Region of China and described the genotypic-phenotypic relationship with *TPO* mutation. A literature study suggests that mutations in the *TPO* gene are one of the most common causes of ID and CH in the Pakistani population^13^. Families with segregating mutations in these genes should be counseled either for genetic or blood-based screening, and preventive therapies should be applied. Recent developments in DNA sequencing technology such as NGS can improve the diagnostic toolset that might help to identify novel causes for CH and related disorders. In general, such data will be helpful in diagnosing and predicting CH/goitrous hypothyroidism, and inborn screening of *TPO* gene mutations will be valuable for the identification of affected newborns or gene carriers in families.

## Supplementary information

Supplementary table 1 (S1)

Supplementary table 2 (S2)

Supplementary table 3 (S3)

Supplementary table 4 (S4)

## Data Availability

The relevant data from this Data Report are hosted at the Human Genome Variation Database at 10.6084/m9.figshare.hgv.2942

## Data availability

The datasets supporting the conclusions of this article are included within the article and its additional file.

## References

[1] Fu C (2016). Mutation screening of the TPO gene in a cohort of 192 Chinese patients with congenital hypothyroidism. BMJ Open.

[2] Macchia PE (1998). PAX8 mutations associated with congenital hypothyroidism caused by thyroid dysgenesis. Nat. Genet..

[3] Medeiros-Neto G., Knobel M., DeGroot L. J. in *Genetics in Endocrinology* (ed. Baxter J. D.) 375–402 (Williams & Wilkins, Philadelphia, Lippincott, 2002).

[4] Castanet M (2001). AFDPHE (Association Francaise pour le Depistage et la Prevention des Handicaps de l’Enfant). Nineteen years of national screening for congenital hypothyroidism: familial cases with thyroid dysgenesis suggest the involvement of genetic factors. J. Clin. Endocrinol. Metab..

[5] Khan A (2019). Homozygous missense variant in the TTN gene causing autosomal recessive limb-girdle muscular dystrophy type 10. BMC Med. Genet..

[6] Wang R, Khan A, Han S, Zhang X (2017). Molecular analysis of 23 Pakistani families with autosomal recessive primary microcephaly using targeted next-generation sequencing. J. Hum. Genet..

[7] Richards S, ACMG Laboratory Quality Assurance Committee. (2015). Standards and guidelines for the interpretation of sequence variants: a joint consensus recommendation of the American College of Medical Genetics and Genomics and the Association for Molecular Pathology. Genet. Med..

[8] Goicoechea de Jorge E (2013). Dimerization of complement factor H-related proteins modulates complement activation in vivo. Proc. Natl Acad. Sci. USA.

[9] Umair M (2019). Biallelic missense mutation in the ECEL1 underlies distal arthrogryposis type 5 (DA5D). Front. Pediatr..

[10] Cangül H, Doğan M, Üstek D (2015). A homozygous nonsense thyroid peroxidase mutation (R540X) consistently causes congenital hypothyroidism in two siblings born to a consanguineous family. J. Clin. Res. Pediatr. Endocrinol..

[11] Pannain S (1999). Two different mutations in the thyroid peroxidase gene of a large inbred Amish kindred: power and limits of homozygosity mapping. J. Clin. Endocrinol. Metab..

[12] Rodrigues C (2005). Mutation screening of the thyroid peroxidase gene in a cohort of 55 Portuguese patients with congenital hypothyroidism. Eur. J. Endocrinol..

[13] Mittal K (2016). Mutations in the genes for thyroglobulin and thyroid peroxidase cause thyroid dyshormonogenesis and autosomal-recessive intellectual disability. J. Hum. Genet..

