# Supplementary material for: Novel homozygous variant in the TPO gene associated with congenital hypothyroidism and mild-intellectual disability
Source: Hum Genome Var. 2020 Nov 27;7:41. doi: 10.1038/s41439-020-00129-3 (PMC7695822; doi:10.1038/s41439-020-00129-3)
Supplement: Supplementary file 1 — Supplementary table 1 (S1) [file 41439_2020_129_MOESM1_ESM.docx]

**Supplementary table 1 (S1):** Detail information about the filtration steps and the screening of candidate variant.

| **Filtration Steps** | **Assessments** |
| --- | --- |
| **Step 1** | Filtering of homozygous variants in the homozygous regions identified after whole genome SNP array |
| **Step 2** | Filtering disease causing variants in already known gene causing a particular disorder from online mendelian inheritance in man(OMIM) and human gene mutation database(HGMD) |
| **Step 3** | Based on identification of novel homozygous or compound heterozygous variants in the genes involved in development,growth,and any pathways involved in CH. |
| **Step 4** | All the identified variants having minor allele frequency (MAF)<0.1% were removed in public SNP databases;1000Genomes (htt://www.1000genomes.org) , ExAC (<http://www.exac.broadinstitute.org>), Hap Map (htt://www.hapmap.ncbi.nlm.nih.gov), Exome Variant Server (<https://evs.gs.washington.edu/EVS/>) , gnomAD (<https://gnomad.broadinstitute.org/>). |
| **Step 5** | Deleterious effects on the function and structure of the protein was identified using SIFT (<http://sift.jcvi.org>), Polyphen-2 (<http://genetics.bwh.harvard.edu/pph2>), mutation taster (<http://mutationtaster.org>), Exome Sequencing Project (ESP, <http://evs.gs.washington.edu/EVS/>), Protein Variation Effect Analyzer (PROVEAN, http://www.provean.jcvi.org), and Combined Annotation Dependent Depletion (CADD, <https://cadd.gs.washington.edu/>). |
| **Step 6** | Thus, the filtered variants were subjected to Sanger sequencing to confirm the co-segregation of the variants with the disease phenotypes within the family. |
