# Supplementary material for: Novel homozygous variant in the TPO gene associated with congenital hypothyroidism and mild-intellectual disability
Source: Hum Genome Var. 2020 Nov 27;7:41. doi: 10.1038/s41439-020-00129-3 (PMC7695822; doi:10.1038/s41439-020-00129-3)
Supplement: Supplementary file 2 — Supplementary table 2 (S2) [file 41439_2020_129_MOESM2_ESM.docx]

**Supplementary Table 2 (S2):** Filtering steps followed to search for the candidate disease causing variant.

| **Filtration methods** | **Number of Variants detected in present family** |
| --- | --- |
| Total variants detected in affected individual | 76,773 |
| Total heterozygous variants detected | 43,934 |
| Total homozygous variants detected | 28,973 |
| Total variants after dbSNPs exclusion | 2,895 |
| Total homozygous frameshift variants detected | 140 |
| Total homozygous indels detected | 122 |
| Total homozygous missense variants detected | 70 |
| Total homozygous nonsense variants detected | 5 |
| Total homozygous splice site variants detected | 68 |
| Total homozygous near splice site variants detected | 18 |
| Total homozygous synonymous variants detected | 34 |
| Total homozygous unknown variants detected | 16 |
| Total homozygous 3’ and 5’ UTR variants detected | 310 |
| Total homozygous variants identified after applying different filters (NHLBI-ESP; 1000 Genomes; ExAC) with MAF>0.01 | 76 |
| Total compound heterozygous variants identified after applying different filters (NHLBI-ESP; 1000 Genomes; ExAC) with MAF>0.01 | 111 |
| Homozygous variant identified in Congenital Hypothyroidism (CH) and intellectual disability (ID) known gene (TPO) and segregating with the disease phenotype in the family | 1 |
