# Supplementary material for: Novel homozygous variant in the TPO gene associated with congenital hypothyroidism and mild-intellectual disability
Source: Hum Genome Var. 2020 Nov 27;7:41. doi: 10.1038/s41439-020-00129-3 (PMC7695822; doi:10.1038/s41439-020-00129-3)
Supplement: Supplementary file 3 — Supplementary table 3 (S3) [file 41439_2020_129_MOESM3_ESM.docx]

**Supplementary table 3 (S3):** List of Known gene for congenital hypothyroidism.

| **S.No** | **Gene name** | **NM_ Number** |
| --- | --- | --- |
| 1 | *TPO* | NM_000547 |
| 2 | *SLC5A5* | NM_000453 |
| 3 | *TG* | NM_003235 |
| 4 | *TSHR* | NM_000369 |
| 5 | *DUOX2* | NM_014080 |
| 6 | *DUOXA2* | NM_207581 |
| 7 | *SLC26A4* | NM_000441 |
| 8 | *FOXE1* | NM_004473 |
| 9 | *PAX8* | NM_013952 |
| 10 | *NKX2-1* | NM_001079668 |
| 11 | *NKX2-5* | NM_004387 |
| 12 | *IYD* | NM_001164694 |
| 13 | *DIO1* | NM_000792 |
| 14 | *DIO2* | NM_000793 |
| 15 | *THRA* | NM_001190918 |
| 16 | *THRB* | NM_00125263 |
| 17 | *DUOX1* | NM_017434 |
| 18 | *DUOXA1* | NM_001276268 |
| 19 | *GNAS* | NM_016592 |
| 20 | *SLC16A2* | NM_006517 |
| 21 | *HHEX* | NM_002729 |
