# Supplementary material for: Novel homozygous variant in the TPO gene associated with congenital hypothyroidism and mild-intellectual disability
Source: Hum Genome Var. 2020 Nov 27;7:41. doi: 10.1038/s41439-020-00129-3 (PMC7695822; doi:10.1038/s41439-020-00129-3)
Supplement: Supplementary file 4 — Supplementary table 4 (S4) [file 41439_2020_129_MOESM4_ESM.docx]

**Table 4.** HGMD reported mutations in *TPO* gene with associated disorders.

| **Gene Name** | **Chromosome**  **Location** | **Transcript ID** | **DNA Variation** | **Protein Variation** | **Mutation Type** | **HGMD Reported phenotype** |
| --- | --- | --- | --- | --- | --- | --- |
| ***TPO*** | **2p25.3** | **NM_000547.5** | **c.2315A>G** | **p.Tyr772Cys** | **Missense** | **Hypothyroidism and ID (This study)** |
| ***TPO*** | 2p25.3 | NM_000547.5 | c.13G>A | p.Ala5Thr | Missense | Thyroid dyshormonogenesis |
| ***TPO*** | 2p25.3 | NM_000547.5 | c.157G>C | p.Ala53Pro | Missense | Total iodide organification defect |
| ***TPO*** | 2p25.3 | NM_000547.5 | c.165C>A | p.Tyr55Term | Nonsense | Hypothyroidism |
| ***TPO*** | 2p25.3 | NM_000547.5 | c.208C>G | p.Pro70Ala | Missense | Hypothyroidism |
| ***TPO*** | 2p25.3 | NM_000547.5 | c.265C>T | p.Arg89Term | Nonsense | Fetal hyperthyroidism |
| ***TPO*** | 2p25.3 | NM_000547.5 | c.281T>C | p.Met94Thr | Missense | Hypothyroidism |
| ***TPO*** | 2p25.3 | NM_000547.5 | c.289T>C | p.Ser97Pro | Missense | Hypothyroidism |
| ***TPO*** | 2p25.3 | NM_000547.5 | c.290C>G | p.Ser97Term | Nonsense | Hypothyroidism |
| ***TPO*** | 2p25.3 | NM_000547.5 | c.391T>C | p.Ser131Pro | Missense | Hypothyroidism |
| ***TPO*** | 2p25.3 | NM_000547.5 | c.404C>A | p.Pro135His | Missense | Hypothyroidism |
| ***TPO*** | 2p25.3 | NM_000547.5 | c.443C>T | p.Ala148Val | Missense | Hypothyroidism |
| ***TPO*** | 2p25.3 | NM_000547.5 | c.502G>A | p.Ala168Thr | Missense | Thyroid dyshormonogenesis |
| ***TPO*** | 2p25.3 | NM_000547.5 | c.524G>A | p.Arg175Gln | Missense | Thyroid peroxidase deficiency |
| ***TPO*** | 2p25.3 | NM_000547.5 | c.523C>T | p.Arg175Term | Nonsense | Thyroid peroxidase deficiency |
| ***TPO*** | 2p25.3 | NM_000547.5 | c.566G>A | p.Arg189Gln | Missense | Hypothyroidism |
| ***TPO*** | 2p25.3 | NM_000547.5 | c.664G>T | p.Asp222Tyr | Missense | Hypothyroidism |
| ***TPO*** | 2p25.3 | NM_000547.5 | c.703C>T | p.Gln235Term | Nonsense | Hypothyroidism |
| ***TPO*** | 2p25.3 | NM_000547.5 | c.718G>A | p.Asp240Asn | Missense | Thyroid peroxidase deficiency |
| ***TPO*** | 2p25.3 | NM_000547.5 | c.719A>T | p.Asp240Val | Missense | Hypothyroidism |
| ***TPO*** | 2p25.3 | NM_000547.5 | c.796C>T | p.Gln266Term | Nonsense | Goitrous hypothyroidism |
| ***TPO*** | 2p25.3 | NM_000547.5 | c.872G>A | p.Arg291His | Missense | Hypothyroidism |
| ***TPO*** | 2p25.3 | NM_000547.5 | c.875C>T | p.Ser292Phe | Missense | Hypothyroidism |
| ***TPO*** | 2p25.3 | NM_000547.5 | c.920A>C | p.Asn307Thr | Missense | Thyroid peroxidase deficiency |
| ***TPO*** | 2p25.3 | NM_000547.5 | c.940C>T | p.Arg314Trp | Missense | Hypothyroidism |
| ***TPO*** | 2p25.3 | NM_000547.5 | c.943C>T | p.Gln315Term | Nonsense | Thyroid peroxidase deficiency |
| ***TPO*** | 2p25.3 | NM_000547.5 | c.955G>A | p.Gly319Arg | Missense | Hypothyroidism |
| ***TPO*** | 2p25.3 | NM_000547.5 | c.961A>G | p.Thr321Ala | Missense | Hypothyroidism |
| ***TPO*** | 2p25.3 | NM_000547.5 | c.962C>T | p.Thr321Ile | Missense | Hypothyroidism |
| ***TPO*** | 2p25.3 | NM_000547.5 | c.976G>A | p.Ala326Thr | Missense | Hypothyroidism |
| ***TPO*** | 2p25.3 | NM_000547.5 | c.992G>T | p.Gly331Val | Missense | Hypothyroidism |
| ***TPO*** | 2p25.3 | NM_000547.5 | c.1022G>A | p.Arg341Gln | Missense | Thyroid dyshormonogenesis |
| ***TPO*** | 2p25.3 | NM_000547.5 | c.1042G>A | p.Gly348Arg | Missense | Hypothyroidism |
| ***TPO*** | 2p25.3 | NM_000547.5 | c.1082G>T | p.Arg361Leu | Missense | Hypothyroidism |
| ***TPO*** | 2p25.3 | NM_000547.5 | c.1117G>T | p.Ala373Ser | Missense | Thyroid dyshormonogenesis |
| ***TPO*** | 2p25.3 | NM_000547.5 | c.1132G>A | p.Glu378Lys | Missense | Goitrous hypothyroidism |
| ***TPO*** | 2p25.3 | NM_000547.5 | c.1152G>T | p.Glu384Asp | Missense | Partial iodide organification defect |
| ***TPO*** | 2p25.3 | NM_000547.5 | c.1159G>A | p.Gly387Arg | Missense | Total iodide organification defect |
| ***TPO*** | 2p25.3 | NM_000547.5 | c.1186C>T | p.Arg396Cys | Missense | Thyroid dyshormonogenesis |
| ***TPO*** | 2p25.3 | NM_000547.5 | c.1193G>C | p.Ser398Thr | Missense | Thyroid dyshormonogenesis |
| ***TPO*** | 2p25.3 | NM_000547.5 | c.1219C>T | p.His407Tyr | Missense | Hypothyroidism |
| ***TPO*** | 2p25.3 | NM_000547.5 | c.1235G>A | p.Arg412His | Missense | ID & thyroid dyshormonogenesis |
| ***TPO*** | 2p25.3 | NM_000547.5 | c.1274A>G | p.Asn425Ser | Missense | Hypothyroidism |
| ***TPO*** | 2p25.3 | NM_000547.5 | c.1277C>G | p.Ala426Gly | Missense | Hypothyroidism |
| ***TPO*** | 2p25.3 | NM_000547.5 | c.1297G>A | p.Val433Met | Missense | Thyroid peroxidase deficiency |
| ***TPO*** | 2p25.3 | NM_000547.5 | c.1313G>A | p.Arg438His | Missense | Hypothyroidism |
| ***TPO*** | 2p25.3 | NM_000547.5 | c.1315A>G | p.Lys439Glu | Missense | Hypothyroidism |
| ***TPO*** | 2p25.3 | NM_000547.5 | c.1327G>C | p.Ala443Pro | Missense | Hypothyroidism |
| ***TPO*** | 2p25.3 | NM_000547.5 | c.1338G>C | p.Gln446His | Missense | Hypothyroidism |
| ***TPO*** | 2p25.3 | NM_000547.5 | c.1339A>T | p.Ile447Phe | Missense | Hypothyroidism |
| ***TPO*** | 2p25.3 | NM_000547.5 | c.1357T>G | p.Tyr453Asp | Missense | Thyroid peroxidase deficiency |
| ***TPO*** | 2p25.3 | NM_000547.5 | c.1373T>C | p.Leu458Pro | Missense | Hypothyroidism |
| ***TPO*** | 2p25.3 | NM_000547.5 | c.1449C>A | p.Asn483Lys | Missense | Hypothyroidism |
| ***TPO*** | 2p25.3 | NM_000547.5 | c.1450G>A | p.Val484Met | Missense | Hypothyroidism |
| ***TPO*** | 2p25.3 | NM_000547.5 | c.1465G>A | p.Ala489Thr | Missense | Hypothyroidism |
| ***TPO*** | 2p25.3 | NM_000547.5 | c.1471C>T | p.Arg491Cys | Missense | Hypothyroidism |
| ***TPO*** | 2p25.3 | NM_000547.5 | c.1472G>A | p.Arg491His | Missense | Hypothyroidism |
| ***TPO*** | 2p25.3 | NM_000547.5 | c.1477G>A | p.Gly493Ser | Missense | Thyroid peroxidase deficiency |
| ***TPO*** | 2p25.3 | NM_000547.5 | c.1483G>A | p.Ala495Thr | Missense | Hypothyroidism |
| ***TPO*** | 2p25.3 | NM_000547.5 | c.1496C>T | p.Pro499Leu | Missense | Thyroid peroxidase deficiency |
| ***TPO*** | 2p25.3 | NM_000547.5 | c.1502T>G | p.Val501Gly | Missense | Goitrous hypothyroidism |
| ***TPO*** | 2p25.3 | NM_000547.5 | c.1535C>A | p.Pro512His | Missense | Hypothyroidism |
| ***TPO*** | 2p25.3 | NM_000547.5 | c.1581G>T | p.Trp527Cys | Missense | Hypothyroidism |
| ***TPO*** | 2p25.3 | NM_000547.5 | c.1597G>T | p.Gly533Cys | Missense | Partial iodide organification defect |
| ***TPO*** | 2p25.3 | NM_000547.5 | c.1618C>T | p.Arg540Term | Nonsense | Thyroid peroxidase deficiency |
| ***TPO*** | 2p25.3 | NM_000547.5 | c.1682C>T | p.Thr561Met | Missense | Hypothyroidism |
| ***TPO*** | 2p25.3 | NM_000547.5 | c.1690C>A | p.Leu564Ile | Missense | Partial iodide organification |
| ***TPO*** | 2p25.3 | NM_000547.5 | c.1727C>T | p.Ala576Val | Missense | Goitrous hypothyroidism |
| ***TPO*** | 2p25.3 | NM_000547.5 | c.1751G>A | p.Arg584Gln | Missense | Hypothyroidism |
| ***TPO*** | 2p25.3 | NM_000547.5 | c.1750C>T | p.Arg584Trp | Missense | Hypothyroidism |
| ***TPO*** | 2p25.3 | NM_000547.5 | c.1759G>A | p.Gly587Arg | Missense | Hypothyroidism |
| ***TPO*** | 2p25.3 | NM_000547.5 | c.1784G>A | p.Arg595Lys | Missense | Goitrous hypothyroidism |
| ***TPO*** | 2p25.3 | NM_000547.5 | c.1784G>C | p.Arg595Thr | Missense | Hypothyroidism |
| ***TPO*** | 2p25.3 | NM_000547.5 | c.1786G>T | p.Glu596Term | Nonsense | Thyroid dyshormonogenesis |
| ***TPO*** | 2p25.3 | NM_000547.5 | c.1858G>A | p.Asp620Asn | Missense | Thyroid dyshormonogenesis |
| ***TPO*** | 2p25.3 | NM_000547.5 | c.1897G>A | p.Asp633Asn | Missense | Thyroid dyshormonogenesis |
| ***TPO*** | 2p25.3 | NM_000547.5 | c.1898A>T | p.Asp633Val | Missense | Hypothyroidism |
| ***TPO*** | 2p25.3 | NM_000547.5 | c.1921G>A | p.Glu641Lys | Missense | Thyroid dyshormonogenesis |
| ***TPO*** | 2p25.3 | NM_000547.5 | c.1943G>A | p.Arg648Gln | Missense | Hypothyroidism |
| ***TPO*** | 2p25.3 | NM_000547.5 | c.1970T>C | p.Ile657Thr | Missense | Hypothyroidism |
| ***TPO*** | 2p25.3 | NM_000547.5 | c.1978C>G | p.Gln660Glu | Missense | Goiter |
| ***TPO*** | 2p25.3 | NM_000547.5 | c.1994G>A | p.Arg665Gln | Missense | Partial iodide organification defect |
| ***TPO*** | 2p25.3 | NM_000547.5 | c.1993C>T | p.Arg665Trp | Missense | Hypothyroidism |
| ***TPO*** | 2p25.3 | NM_000547.5 | c.2000G>A | p.Gly667Asp | Missense | Goitrous hypothyroidism |
| ***TPO*** | 2p25.3 | NM_000547.5 | c.1999G>A | p.Gly667Ser | Missense | Thyroid peroxidase deficiency |
| ***TPO*** | 2p25.3 | NM_000547.5 | c.2017G>A | p.Glu673Lys | Missense | Hypothyroidism |
| ***TPO*** | 2p25.3 | NM_000547.5 | c.2059G>T | p.Glu687Term | Nonsense | Hypothyroidism |
| ***TPO*** | 2p25.3 | NM_000547.5 | c.2077C>T | p.Arg693Trp | Missense | Hypothyroidism |
| ***TPO*** | 2p25.3 | NM_000547.5 | c.2101C>T | p.Leu701Phe | Missense | Hypothyroidism |
| ***TPO*** | 2p25.3 | NM_000547.5 | c.2173A>C | p.Thr725Pro | Missense | Hypothyroidism |
| ***TPO*** | 2p25.3 | NM_000547.5 | c.2242G>A | p.Val748Met | Missense | Hypothyroidism |
| ***TPO*** | 2p25.3 | NM_000547.5 | c.2266T>C | p.Cys756Arg | Missense | Goitrous hypothyroidism |
| ***TPO*** | 2p25.3 | NM_000547.5 | c.2305C>T | p.Arg769Trp | Missense | Thyroid dyshormonogenesis |
| ***TPO*** | 2p25.3 | NM_000547.5 | c.2311G>A | p.Gly771Arg | Missense | Hypothyroidism |
| ***TPO*** | 2p25.3 | NM_000547.5 | c.2327G>A | p.Gly776Asp | Missense | Hypothyroidism |
| ***TPO*** | 2p25.3 | NM_000547.5 | c.2395G>A | p.Glu799Lys | Missense | Thyroid peroxidase deficiency |
| ***TPO*** | 2p25.3 | NM_000547.5 | c.2398T>C | p.Cys800Arg | Missense | Goitrous hypothyroidism |
| ***TPO*** | 2p25.3 | NM_000547.5 | c.2422T>C | p.Cys808Arg | Missense | Thyroid peroxidase deficiency |
| ***TPO*** | 2p25.3 | NM_000547.5 | c.2512T>A | p.Cys838Ser | Missense | Hypothyroidism |
| ***TPO*** | 2p25.3 | NM_000547.5 | c.2515G>A | p.Val839Ile | Missense | Hypothyroidism |
| ***TPO*** | 2p25.3 | NM_000547.5 | c.2578G>A | p.Gly860Arg | Missense | Thyroid peroxidase deficiency |
| ***TPO*** | 2p25.3 | NM_000547.5 | c.2587G>A | p.Ala863Thr | Missense | Hypothyroidism |
| ***TPO*** | 2p25.3 | NM_000547.5 | c.2599T>C | p.Ser867Pro | Missense | Thyroid dyshormonogenesis |
| ***TPO*** | 2p25.3 | NM_000547.5 | c.2619G>A | p.Trp873Term | Nonsense | Hypothyroidism |
| ***TPO*** | 2p25.3 | NM_000547.5 | c.2647C>T | p.Pro883Ser | Missense | Hypothyroidism |
| ***TPO*** | 2p25.3 | NM_000547.5 | c.2665G>A | p.Gly889Arg | Missense | Thyroid dyshormonogenesis |
| ***TPO*** | 2p25.3 | NM_000547.5 | c.2665G>T | p.Gly889Term | Nonsense | Hypothyroidism |
| ***TPO*** | 2p25.3 | NM_000547.5 | c.2717C>T | p.Pro906Leu | Missense | Hypothyroidism |
| ***TPO*** | 2p25.3 | NM_000547.5 | c.2749G>A | p.Glu917Lys | Missense | Hypothyroidism |
| ***TPO*** | 2p25.3 | NM_000547.5 | c.349G>C | p.Asp117His | Missense | Hypothyroidism |
| ***TPO*** | 2p25.3 | NM_000547.5 | c.483-2A>G | - | Splice site | Hypothyroidism |
| ***TPO*** | 2p25.3 | NM_000547.5 | c.820-2A>G | - | Splice site | Hypothyroidism |
| ***TPO*** | 2p25.3 | NM_000547.5 | c.1339A>T | - | Splice site | Hypothyroidism |
| ***TPO*** | 2p25.3 | NM_000547.5 | c.1597+1G>T | - | Splice site | Partial iodide organification defect |
| ***TPO*** | 2p25.3 | NM_000547.5 | c.1768G>A | p.Gly590Ser | missense | Thyroid peroxidase deficiency |
| ***TPO*** | 2p25.3 | NM_000547.5 | c.1768+1G>A | - | Splice site | Hypothyroidism |
| ***TPO*** | 2p25.3 | NM_000547.5 | c.2386G>T | p.Asp796Tyr | missense | Thyroid peroxidase deficiency |
| ***TPO*** | 2p25.3 | NM_000547.5 | c.2386+2T>G | - | Splice site | Hypothyroidism |
| ***TPO*** | 2p25.3 | NM_000547.5 | c.2618+1G>T | - | Splice site | Hypothyroidism |
| ***TPO*** | 2p25.3 | NM_000547.5 | c.2748G>A |  | Splice site | Hypothyroidism |
| ***TPO*** | 2p25.3 | NM_000547.5 | c.-80A>G | - | Regulatory sequence | Decreased thyroid mRNA expression |
| ***TPO*** | 2p25.3 | NM_000547.5 | c.215delA | p.Gln72Argfs*15 | Frameshift | Total iodide organification defect |
| ***TPO*** | 2p25.3 | NM_000547.5 | c.387delC | p.Asn129Lysfs*80 | Frameshift | Thyroid peroxidase deficiency |
| ***TPO*** | 2p25.3 | NM_000547.5 | c.477delC | p.Asn159Lysfs*50 | Frameshift | Total iodide organification defect |
| ***TPO*** | 2p25.3 | NM_000547.5 | c.669_671delTGA | p.Asp224del | Frameshift | Hypothyroidism |
| ***TPO*** | 2p25.3 | NM_000547.5 | c.670_672delGAC | p.Asp224del | Frameshift | Thyroid dyshormonogenesis |
| ***TPO*** | 2p25.3 | NM_000547.5 | c.843delC | p.Ala282Argfs*36 | Frameshift | Thyroid peroxidase deficiency |
| ***TPO*** | 2p25.3 | NM_000547.5 | c.1237delG | p.Glu413Serfs*38 | Frameshift | Thyroid dyshormonogenesis |
| ***TPO*** | 2p25.3 | NM_000547.5 | c.1336delC | p.Gln446Argfs*5 | Frameshift | Hypothyroidism |
| ***TPO*** | 2p25.3 | NM_000547.5 | c.1496delC | p.Pro499Argfs*3 | Frameshift | Goitrous hypothyroidism |
| ***TPO*** | 2p25.3 | NM_000547.5 | c.1721_1726delATCTGG | p.Asp574_Leu575del | Frameshift | Partial iodide organification defect |
| ***TPO*** | 2p25.3 | NM_000547.5 | c.1851delC | p.Ser617Argfs*23 | Frameshift | Hypothyroidism |
| ***TPO*** | 2p25.3 | NM_000547.5 | c.2153_2154delTT | p.Phe718* | Frameshift | Hypothyroidism |
| ***TPO*** | 2p25.3 | NM_000547.5 | c.2243delT | p.Val748Glyfs*50 | Frameshift | Total iodide organification defect |
| ***TPO*** | 2p25.3 | NM_000547.5 | c.2413delC | p.His805Thrfs*27 | Frameshift | Thyroid peroxidase deficiency |
| ***TPO*** | 2p25.3 | NM_000547.5 | c.2421delC | p.Cys808Alafs*24 | Frameshift | Hypothyroidism |
| ***TPO*** | 2p25.3 | NM_000547.5 | c.2422delT | p.Cys808Alafs*24 | Frameshift | Hypothyroidism |
| ***TPO*** | 2p25.3 | NM_000547.5 | c.2619-5_2623del10 | - | Frameshift | Goitrous hypothyroidism |
| ***TPO*** | 2p25.3 | NM_000547.5 | c.2622_2638del17 | p.Arg875Hisfs*100 | Frameshift | Hypothyroidism |
| ***TPO*** | 2p25.3 | NM_000547.5 | c.2723_2732del10 | p.Arg908Leufs*63 | Frameshift | Hypothyroidism |
| ***TPO*** | 2p25.3 | NM_000547.5 | c.2738_2748+5del16 | - | Frameshift | Hypothyroidism |
| ***TPO*** | 2p25.3 | NM_000547.5 | c.31_50dup20 | p.Glu17Aspfs*77 | Frameshift | Hypothyroidism |
| ***TPO*** | 2p25.3 | NM_000547.5 | c.1184_1187dupGCCG | p.Ala397Profs*76 | Frameshift | Goiter |
| ***TPO*** | 2p25.3 | NM_000547.5 | c.1696dupG | p.Val566Glyfs*28 | Frameshift | Hypothyroidism |
| ***TPO*** | 2p25.3 | NM_000547.5 | c.1955dupT | p.Phe653Valfs*16 | Frameshift | Goitrous hypothyroidism |
| ***TPO*** | 2p25.3 | NM_000547.5 | c.2268dupT | p.Glu757* | Frameshift | Total iodide organification defect |
| ***TPO*** | 2p25.3 | NM_000547.5 | c.2421dupC | p.Cys808Leufs*72 | Frameshift | Hypothyroidism |
| ***TPO*** | 2p25.3 | NM_000547.5 | c.1188_1193delCGCCAGins10 | p.Ala397Profs*76 | Frameshift | Hypothyroidism |
| ***TPO*** | 2p25.3 | NM_000547.5 | 1519_1539del | p. A477_N483del | Frameshift | Thyroid peroxidase deficiency |
| ***TPO*** | 2p25.3 | NM_000547.5 | c.1529_1564 | - | Frameshift | Hypothyroidism |
| ***TPO*** | 2p25.3 | NM_000547.5 | ex. 11-15del | - | Frameshift | ID |

HGMD; Human Genome Mutation Database, ID; Intellectual Disability, ex; Exon
